# Pathological vertebral fracture after stereotactic body radiation therapy for lung metastases. Case report and literature review.

**DOI:** 10.1186/1748-717X-7-50

**Published:** 2012-03-28

**Authors:** María Esperanza Rodríguez-Ruiz, Iñigo San Miguel, Ignacio Gil-Bazo, Jose Luis Perez-Gracia, Leire Arbea, Marta Moreno-Jimenez, Javier Aristu

**Affiliations:** 1 Radiation Oncology Department, University of Navarra Clinic, Avda Pio XII, 36, C.P, 31008 Pamplona, Spain; 2 Radiation Oncology Department, University of Navarra Clinic, Pamplona, Spain; 3 Oncology Department, University of Navarra Clinic, Pamplona, Spain

**Keywords:** Stereotactic Body Radiation therapy, Adverse effects, Pathological vertebral fracture

## Abstract

**Background:**

Stereotactic body radiation therapy (SBRT) is a radiation technique used in patients with oligometastatic lung disease. Lung and chest wall toxicities have been described in the patients but pathological vertebral fracture is an adverse effect no reported in patients treated with SBRT for lung metastases.

**Case presentation:**

A 68-year-old woman with the diagnosis of a recurrence of a single lung metastatic nodule of urothelial carcinoma after third line of chemotherapy. The patient received a hypo-fractionated course of SBRT.A 3D-conformal multifield technique was used with six coplanar and one non-coplanar statics beams. A total dose of 48 Gy in three fractions over six days was prescribed to the 95% of the CTV. Ten months after the SBRT procedure, a CT scan showed complete response of the metastatic disease without signs of radiation pneumonitis. However, rib and vertebral bone toxicities were observed with the fracture-collapse of the 7^th ^and 8^th ^vertebral bodies and a fracture of the 7^th ^and 8^th ^left ribs. We report a unique case of pathological vertebral fracture appearing ten months after SBRT for an asymptomatic growing lung metastases of urothelial carcinoma.

**Conclusion:**

Though SBRT allows for minimization of normal tissue exposure to high radiation doses SBRT tolerance for vertebral bone tissue has been poorly evaluated in patients with lung tumors. Oncologists should be alert to the potential risk of fatal bone toxicity caused by this novel treatment. We recommend BMD testing in all woman over 65 years old with clinical risk factors that could contribute to low BMD. If low BMD is demonstrated, we should carefully restrict the maximum radiation dose in the vertebral body in order to avoid intermediate or low radiation dose to the whole vertebral body.

## Background

Stereotactic body radiation therapy (SBRT) is a new external radiation treatment modality that involves the precise irradiation of extracranial tumors using very high doses of radiation in one to five fractions. SBRT has been used mainly to treat inoperable lung and liver tumors, but it is also used in the treatment of oligometastatic lung tumors [1]. Phase I/II data from patients with oligometastatic lung disease who were treated with SBRT seem to be very promising, with high rates of local control and long-term survivors in properly selected patients [2,3].

SBRT for metastatic lung disease can produce radiation pneumonitis, but the reported incidence of grade 3-4 pneumonitis is low, in the range of 0-5%. Nonpulmonary toxic effects, such as esophagitis, esophageal stenosis, massive hemoptysis, pleural effusion and pericardial effusion, are more frequently observed in centrally located primary lung tumors when they are treated with a total dose of 60-66 Gy (three fractions of 20 Gy). These toxic effects have not been reported in lung metastatic tumors that are peripherally located, and the presence of pathologic vertebral fractures have only been described in patients who were treated with SBRT for metastatic spinal tumors. We report a unique case of a pathologic vertebral fracture appearing 10 months after SBRT for an asymptomatic growing lung metastasis of urothelial carcinoma.

### Case presentation

A 68-year-old woman was referred to our department in February 2008 for a second opinion regarding a metastatic urothelial carcinoma. Her history began six months prior to her visit when she presented with recurrent urinary tract infections. A cystoscopy and TUR revealed a high grade infiltrating urothelial carcinoma in the lateral right wall of the bladder (T2a). Staging CT scans were performed and showed metastatic disease mediastinal and hiliar lymph nodes, multiple and bilateral lung nodules, and metastases located in the internal iliac lymph nodes. The patient was initially treated with six cycles of chemotherapy with gemcitabine 1200 mg/m^2 ^on day 1 and 60 mg/m^2 ^cisplatin on days 1 and 8 every 21 days, showing a partial response after three cycles. The disease was followed up until January 2009, when imaging studies demonstrated the presence of a single metastatic lesion in the left lower lung lobe. A second line of chemotherapy using carboplatin AUC = 3 on day 1 and 1500 mg/m^2 ^gemcitabine on day 1 every 15 days was initiated, and after eight cycles, new progression of the described lung lesion was documented. A third line of chemotherapy was proposed, and in June 2009, the patient was started on cisplatin 60 mg/m^2 ^and pemetrexed 500 mg/m^2 ^day 1 every 28 days. She had a partial response after two cycles, and after four cycles of chemotherapy, a slight increase in the size of the left lower lung nodule was revealed on the CT scan, although a bone scan was negative.

**Figure 1:**
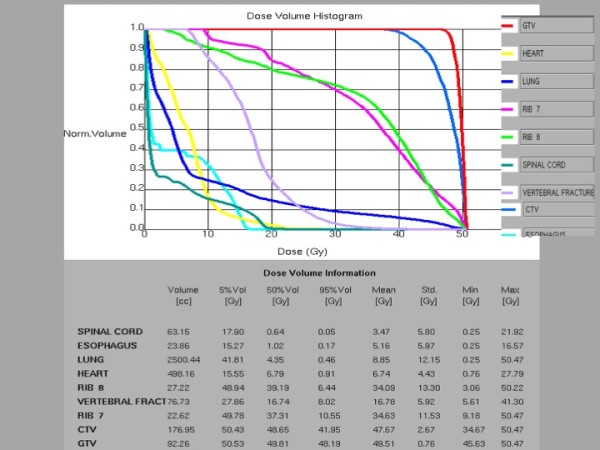
**Dose volume histograms showing dosimetric parameters analyzed including targets and organs at risk**.

With the diagnosis of a recurrence of a single lung metastatic nodule measuring 56 × 53 × 51 millimeters, in October 2009, a hypo-fractionated course of SBRT was delivered to the lung nodule. This patient was placed in a supine position and was immobilized using an alpha cradle device to improve the reproducibility of the setup during daily treatments. A planning CT scan was performed. A combination of three sets of CT scans obtained during free breathing, deep inspiration and deep expiration were used to generate an internal target volume (ITV) that accounts for respiratory motion. The gross tumor volume (GTV) was the lung nodule, and the clinical target volume (CTV) included the ITV with a symmetric 0.5 cm margin. Respiratory tumor movement was observed under fluoroscopy. The delimited organs at risk (OARS) and the applied constraints are listed in Figure 1.

**Figure 2:**
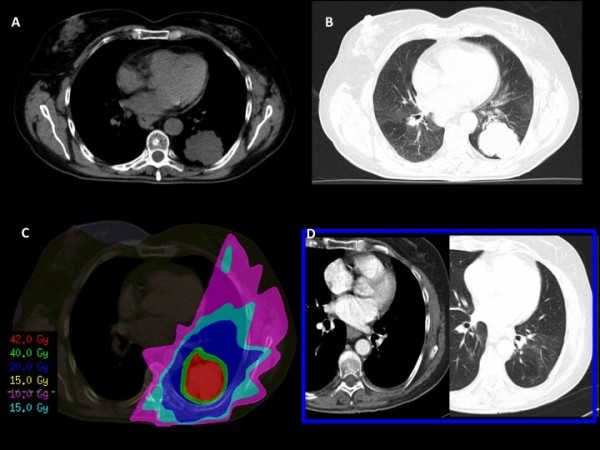
**Lung metastases in the chest CT scan (A and B) before SBRT**. SBRT dose distribution (C) and CT scan six months after SBRT in which complete response of metastatic disease is demonstrated.

A 3D-conformal multifield technique was used with six coplanar and one non-coplanar statics beams. SBRT was delivered by a linear accelerator (Oncor, Siemens, Palo Alto, California, USA) with 6-MV photons. A total dose of 48 Gy in three fractions over six days was prescribed to the 95% of the CTV (Figure 2). Patient positioning and isocenter verification were checked using cone beam CT.

## Results

The patient continued a regular follow-up program with history, physical examination, complete blood count, liver and renal function tests, thorax-CT scan and bone scan were performed at 3-month intervals. Ten months after the SBRT procedure, a CT scan showed complete response of the metastatic disease without signs of radiation pneumonitis and segmental atelectasis. However, rib and vertebral bone toxicities were observed with the fracture-collapse of the 7^th ^and 8^th ^vertebral bodies and a fracture of the 7^th^-8^th ^left ribs. Magnetic resonance imaging (MRI) of the spine was performed and showed vertebral collapse in the top plate of the 7^th ^and 8^th ^vertebral bodies with anterior wedging signs and bone edema in the left pedicle. There was no associated soft tissue mass, spinal cord compression or invasion of the spinal canal. A percutaneous vertebral biopsy showed fibrosis and no tumoral cells were discovered in the pathologic analysis.

**Figure 3:**
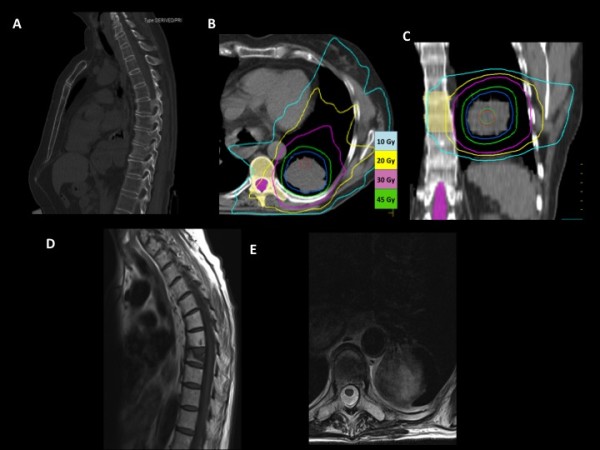
**Sagital view of CT slide before SBRT (A)**. Transverse (B) and coronal (C) view of SBRT dose distribution in the lung metastases. Blue, yellow, and pink lines represent the 10 Gy, 20 Gy and 30 Gy isodose lines, respectively. The 10 Gy isodose distribution shapped the 7^th^-8^th ^vertebral fractures. (D) Sagittal and coronal (E) view of MRI ten moths before SBRT.

**Figure 4:**
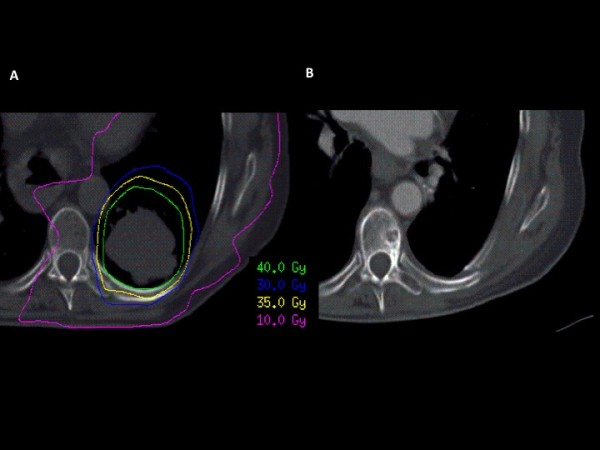
**Radiation dose distribution in the transverse plane (A) and radiation dose distribution co-registered with CT images during the follow-up (B)**.

Reviewing the treatment plan and considering the vertebral MRI, we determined that the vertebral lesion was located marginal to the high radiation dose, noting that the 10 Gy isodose line shaped the 7^th ^and 8^th ^vertebral fracture (Figure 3). However, the dosimetric review showed that the 40 Gy isodose line shapped the 7^th ^and 8^th ^rib fractures and the volume of the chest wall receiving at least 30 Gy was > 35 cc (V30). The Dmax in the fractured ribs was 50 Gy (Figure 4).

At present, the patient continues to be asymptomatic without any evidence of metastatic disease based on physical examination, CT scans and spinal MR images. However, a bone mineral density measurements for lumbar spine (L2-L4 area) with dual energy X-ray absorptiometry were performed and showed a low bone mineral density (BMD) of 0,7346 gr/cm2 which was equivalent to a T score of -3,02; therefore, our patient was diagnosed with osteoporosis.

## Discussion

SBRT delivers large doses of radiation in few fractions, and it has been demonstrated to be a safe and effective radiation technique for the treatment of selected patients with lung metastases. The reported 2-year local control ranges from 67% to 100% [4] and asymptomatic pneumonitis is the most frequently observed grade 1-2 adverse effect. A low incidence of grade 3 pulmonary and nonpulmonary toxicity has been reported in patients with lung metastases treated with SBRT. The most common nonpulmonary toxicities observed in patients with lung metastases treated with SBRT are skin rash and rib fracture. Nevertheless, vertebrae toxicity has only been reported in patients treated with SBRT for spinal tumors[5-8].

Rib fractures can be asymptomatic, and therefore, this finding can be under-reported. Most of the studies with SBRT in lung cancer have related the risk of rib fracture when the volume of chest wall receiving at least 30 Gy (V30) is > 35 cc. A recent combined analysis of patients treated with thoracic SBRT from the University of Virginia and Colorado revealed that the V30 was the best predictor of chest wall toxicity [9]. Our patient presented with asymptomatic rib fractures. Then V30, V40 and Dmax of the fractured ribs were < 35 cc (19 cc), 11 cc and 50 Gy, respectively. However, the largest study reported in the literature related to rib toxicity has been published by Andolino in 2011[10]. This study concluded that to minimize toxicity when treating lesions in close proximity to the chest wall, the Dmax of the chest wall and/or ribs should be < 50 Gy, and < 5 cc of the chest wall should receive ≥ 40 Gy.

Limited data exist on the risk of vertebral fracture after radiation therapy. Pathologic vertebral fracture has not been described in patients treated with SBRT for lung metastases. SBRT on the spine can result in pathologic vertebral fractures, especially when at least 20% of the vertebral body demonstrates metastatic involvement at the time of treatment and when the lesion is lytic [6]. The use of high-dose single-fraction SBRT in patients with spinal metastases has also resulted in a significant rate of new or progressive vertebral compression. In a recent study from Memorial Sloan-Kettering Cancer Center, fracture progression occurred in 27 (39%) of 71 vertebrae treated with single dose SBRT [11]. In fact, the recommended SBRT vertebral bone dose-volume constraints remain an issue that is under investigation. In addition, our patient presented an asymptomatic 7^TH ^and 8^TH ^vertebral body collapse ten months after SBRT, and the 10 Gy isodose curve shaped the morphology of the fracture. The imaging suggests that the pathologic fracture is the result of the exposure of high radiation dose at least in the left half of the vertebrae body. We have reviewed the radiation dose homogeneity in the vertebral body and the SD of the dose distribution was 5.92 Gy and the homogeneity index (D5/D95) was 3.6 Gy. It is very difficult to prove that radiation hot spots in the vertebral body are the cause of the fracture because no data are available in the literature regarding the risk of vertebral fracture and the homogeneity index in patients treated with SBRT. The present clinical observation must take into account other associated causes of pathological vertebral fracture. For example, variables such as age, gender, body size, dietary calcium, regular exercises, drug, caffeine and alcohol consumption, bone density and other metabolic disturbances can contribute to low BMD. The "Clinician's Guide to Prevention and Treatment of Osteoporosis (2010)" developed by the National Osteoporosis Foundation recommends BMD testing in women aged 65 yrs and older, regardless of clinical risk factors [12]. Initially, this 68-years-old postmenopausal woman was not considered to be a candidate for a BMD test before SBRT; this was, because she was evaluated clinically, had no risk factors for osteoporosis other than age, and had been treated with chemotherapy and corticotherapy. After the pathologic vertebral fractures, bone mineral density measurements for the lumbar spine (L2-L4 area) with dual energy X-ray absorptiometry was performed and showed a low BMD of 0.7346 gr/cm2 which was equivalent to a T score of -3,02; therefore, our patients was diagnosed of osteoporosis. Woman with osteoporosis may be an important factor to identify patients with high risk of severe bone toxicity after SBRT and to minimize the risk of fractures we should be more restrictive in the recommended constraints on the chest wall and central structures.

In conclusion, SBRT tolerance for vertebral bone tissue has been poorly evaluated in patients with lung tumors. To the best of our knowledge, the pathologic vertebral fracture in this patient is the first reported using SBRT for lung metastases. Before SBRT, we recommend BMD testing in all woman over 65 years old with clinical risk factors that could contribute to low BMD. If low BMD is demonstrated, we should carefully restrict the maximum radiation dose in the vertebral body in order to avoid intermediate or low radiation dose to the whole vertebral body. More studies are needed to determine the radiation dose tolerance of the vertebral body to prevent vertebral collapse in populations with clinical risk factors of pathologic fractures such as low BMD.

## Consent

Written informed consent was obtained from the patient for the publication of this case report and any accompanying images.

## Competing interests

The authors declare that they have no competing interests.

## Authors' contributions

All the authors contributed to drafting the manuscript and all authors reviewed and approved the final manuscript.
